# Double Burden of Malnutrition among Chinese Women of Reproductive Age and Their Social Determinants

**DOI:** 10.3390/nu12103102

**Published:** 2020-10-12

**Authors:** Jingqi Song, Ji Zhang, Wafaie Fawzi, Yangmu Huang

**Affiliations:** 1 School of Public Health, Peking University Health Science Center, Beijing 100191, China; jqsong30@163.com (J.S.); jizhang@bjmu.edu.cn (J.Z.); 2 Harvard T.H. Chan School of Public Health, Harvard University, Boston, MA 02115, USA; mina@hsph.harvard.edu

**Keywords:** malnutrition, women of reproductive age, determinants

## Abstract

This study aimed to examine the impact of a wide range of demographic, socioeconomic, and community factors on the double burden of malnutrition among women of reproductive age using longitudinal data. We used data about 11,348 women of reproductive age who participated in the China Health and Nutrition Survey (CHNS), a longitudinal survey, between 1989 and 2015. Nutritional outcomes were categorized into four groups, namely underweight, normal weight, overweight, and obesity, with normal weight as reference. A multinomial logit model was fitted due to geographic clustering and repeated observations of individuals. The prevalence of underweight decreased over time from 1991 but has tended to rise again since 2004, while the prevalence of overweight/obesity continued to rise between 1991 and 2015. Improved individual factors, socioeconomic status, and community urbanization reduced the risk of underweight but elevated the risk of overweight and obesity. The medium levels, rather than the highest levels, of household income and community urbanization are associated with a higher risk of overweight and obesity. The notable increase in underweight prevalence is a cause for concern to be addressed along with efforts to curb the rising tide of overweight. In order to enhance the nutritional status of women of reproductive age, it is essential to improving the community environment, levels of education, and living environment from a wider context. Long-term and targeted plans are urgently needed for nutrition improvements among the different populations.

## 1. Introduction

The nutritional status of women of reproductive age has a crucial influence on the health of women in the pre-pregnancy period and has implications for pregnancy outcomes and health for the next generation [1]. It is closely related to nutritional reserves and status throughout pregnancy [2], as well as the development of the fetus and newborn [3]. For instance, about 468 million women aged 15–49 (30% of women’s population) are suffering from anemia worldwide, with the highest proportion in Africa (48–57%) and the largest number of them in Southeast Asia (182 million of childbearing age and 18 million in the state of pregnancy) [4]. Anemia is the second leading cause of maternal death in Asia, accounting for approximately 12.8% of independent deaths from postpartum hemorrhage. Recent studies have shown that about 20% of maternal deaths are caused by anemia, which is a risk factor that accounts for 50% of all maternal deaths [5]. Iron deficiency anemia is the most common type of anemia caused by inadequate iron intake and storage in the body. It is the most common nutritional disorder in the world and is affecting many children and women in developing countries [6]. At the same time, it is essential to note that iron deficiency anemia is usually preventable and highly treatable [7]. Therefore, there is a strong need to pay more attention to women’s nutritional status to decrease the complications resulting from such preventable health disorders. Although the nutritional status of women in low- and middle-income countries (LMICs) has improved over the past decades due to economic growth [8], poor nutrition status (underweight, micronutrient deficiency, etc.) remains a significant public health problem. China is no exception, and poor nutrition and food insecurity are still common in rural or economically undeveloped areas, which may seriously affect an individual’s physical, social, and psychological functions [9]. In addition, over the past two decades, the trend of overweight or obesity among women and adolescents of reproductive age in urban and rural areas has increased [10,11]. Overweight and obesity are considered a major risk factor for chronic diseases, such as cardiovascular disease, hypertension, and type 2 diabetes [12]. This double burden of malnutrition has become a new public health concern for China [13].

Malnutrition denotes undernutrition and overnutrition, both of which could lead to the development of diseases and chronic health conditions if not addressed [14]. Evidence has suggested that the prevalence of unplanned pregnancy has reached 40–50% in European and Latin American countries, especially among adolescents [15]. Meanwhile, unplanned pregnancy, accounting for almost 10% of fertile women each year in China, is associated with missed dietary nutrition improvement during the preconception period [16]. Considering that the first few weeks of pregnancy are a critical period for fetal growth and development, malnutrition could induce adverse outcomes such as small for gestational age birth, preterm birth, low birth weight, congenital anomalies, and stillbirth [17]. It is worthwhile to understand the nutritional status of women of reproductive age and identify the risk factors associated with it. This can contribute to intervention efforts, breaking the cycle of malnutrition and gradually eliminating nutrition-related health problems.

Girma and Genebo conducted a study on the determinants of the nutritional status of women and children in Ethiopia [18]. The study found that socioeconomic and community risk factors were associated with malnutrition. For instance, the region of residence, household economic status, a woman’s employment status, and decision-making power over income are potential causes for women’s chronic energy deficiency (CED) [18], defined as a steady state at which a person is in energy balance at a cost to their health [19]. Meanwhile, another study in China indicated that unfavorable lifestyles, gender, age, ethnicity, education levels, and physical activity could also be the risk factors of overweight [20]. Most of these studies focused on either undernutrition or overweight/obesity instead of both. Samples were restricted to certain age ranges with relatively short study periods. Moreover, suggestions on nutritional intervention were concentrated on the third trimester, and largely overlooked the pre-pregnancy stage or the whole reproductive age period. This study aimed to fill these gaps, focusing on factors influencing malnutrition among Chinese women of reproductive age by using nationwide longitudinal datasets. Data from 1989 to 2015 were used in this study, which aimed to address the potential temporal problems in the analysis.

## 2. Materials and Methods

### 2.1. Dataset and Study Sample

The China Health and Nutrition Survey (CHNS) is an ongoing open cohort of the Chinese population. It adopts a multistage and random cluster process to draw a sample of about 7200 households with over 30,000 individuals in 12 provinces (Liaoning, Jiangsu, Shandong, Henan, Hubei, Hunan, Guangxi, Guizhou, and Heilongjiang) and cities (Beijing, Shanghai, and Chongqing). Cities in China are divided into cities, suburbs, counties, and rural areas according to different socioeconomic levels, and a city was randomly selected in each level based on these four levels. The survey was conducted in 1989, 1991, 1993, 1997, 2000, 2004, 2006, 2009, 2011, and 2015, including individual, household, and community data referring to health, demographic, socioeconomic, and nutrition aspects. All 10 waves of the CHNS data were used in our study. We narrowed the cohort to women of reproductive age (15–49 years) and excluded records for those who were pregnant (*n* = 198) or with missing data of height and weight. In all, 24,606 records were retained after the exclusion, and data from the wave of 1989 was not included in the retained records due to the lack of data about health insurance, smoking, or alcohol consumption. Survey protocols, instruments, and the process for obtaining informed consent for this study were approved by the institutional review committees of the University of North Carolina at Chapel Hill and the National Institute of Nutrition and Food Safety, China Center for Disease Control and Prevention. All participants and/or their parents/guardians provided written informed consent for their participation in the survey.

### 2.2. Outcomes of Interest

The CHNS recorded height and weight for each individual within the household, measured by health professionals. Body mass index (BMI) was calculated as weight divided by height squared (kg/m^2^) and was categorized according to the following World Health Organization (WHO) guidelines for Asian populations: underweight (<18.5 kg/m^2^), normal weight (18.5–22.9 kg/m^2^), overweight (23.0–27.4 kg/m^2^), and obesity (≥27.5 kg/m^2^) [21].

### 2.3. Variables

#### 2.3.1. Individual-Level Factors

Individual-level variables included education level, marital status, nationality (i.e., Han or other), annual household income per capita, and whether the individual had medical insurance. Educational level was determined based on the response to the following question: “What is the highest level of education you have attained?” Participants were divided into the following four groups: illiterate or primary school dropout, junior and primary school graduates, high school or vocational school, and university or above. Marital status was categorized into single, married, and divorced/widowed/separated. In order to control the different purchasing power values across the households, annual household income per capita was categorized into tertiles of low-, medium-, and high-income groups. We also controlled for age as a demographic variable, as well as nationality, which has cultural effects on dietary habits.

#### 2.3.2. Community-Level Factors

Subjects were categorized into regional groups according to the provinces or cities to which they belonged. Province data were grouped based on the following geographical and socioeconomic disparities: coastal (Shanghai, Shandong, and Jiangsu), central (Beijing, Henan, Hubei, and Hunan), northeast (Liaoning and Heilongjiang), and southwest (Chongqing, Guangxi, and Guizhou) regions. The southwest is the most economically deprived region and was set as reference. The region was controlled in order to capture unobserved geographic and cultural factors related to food consumption and food prices. We also included wave variables to capture temporal changes with 1991 as reference.

Additionally, each community has its index as a community-level measure of urbanization, including the level of 12 components as follows: population density, economic activity, traditional markets, modern markets, transportation infrastructure, sanitation, communications, housing, education, diversity, health infrastructure, social services [22]. This variable was included in our models to control for multilevel and multistage sampling and set as an array of multilevel modeling issues. This was important because of the link of malnutrition in the target population with the social and environmental contexts [23]. To control for neighborhood effects on the nutrition status of women of reproductive age, urbanization tertiles were generated to show relative urbanization rankings of each community.

### 2.4. Statistical Analysis

Frequencies and percentages were used to describe the characteristics of the participants. The age of the participants was presented as the mean (standard deviation). Chi-squared tests were used to examine the trends in underweight, overweight, and obesity among women of reproductive age during the years of 1991–2015. Unordered logistic regression modeling was used due to the nominal nature of the dependent variables. To account for clustering, a multilevel approach was adopted to explore the demographic characteristics and risks of malnutrition. Due to the relatively small number of women of reproductive age within the same household on average, we ignored the household level and focused on the community level and individual level. Therefore, the models were fitted with repeated individuals (level 1) and communities (level 2). The multilevel model accounts for the hierarchical structure of data; it can correct biases in parameter estimates and provide accurate regression coefficients (standard errors (SEs)) and produce correct confidence intervals (CIs) and significance tests, thereby handling the clustering of observations that occurs within units [24]. SEs and prospective odds ratios (ORs) (with 95% CIs) in the logistic regression were calculated for BMI level. All data analyses were conducted using Statistical Analysis System (SAS) version 9.4 (SAS Institute Inc., Cary, NC, USA). All *p*-values were two-sided, and *p* < 0.05 was considered to be statistically significant.

### 2.5. Patient and Public Involvement

This research used data from CHNS, which was designed to see how the social and economic transformation of Chinese society is affecting the health and nutritional status of its population. Participants in the original study provided detailed information at the community, household, and individual level, with a physical examination as well at each wave. All participants and/or their parents/guardians provided written informed consent for their participation in the CHNS.

## 3. Results

### 3.1. Malnutrition Prevalence and Baseline Characteristics

**Figure 1:**
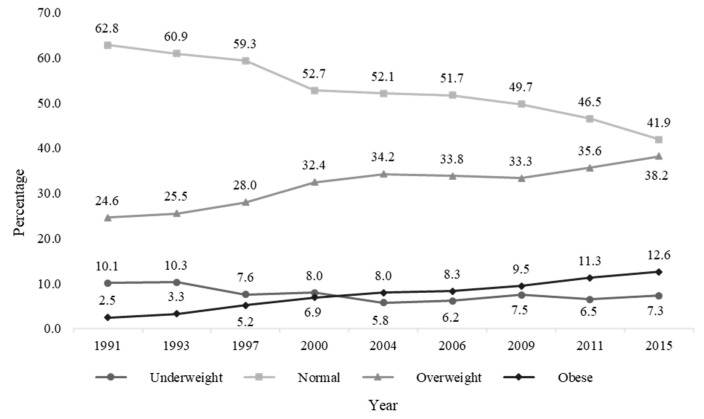
Trends of body mass index (BMI) among women of reproductive age by survey year.

The sample was restricted to complete cases for nutritional outcomes and predictors considered in the analyses. The sample size of women of reproductive age (aged 15–49) was 11,348, clustered in 316 communities with 24,606 observations. The prevalence of underweight among women of reproductive age decreased over time from 10.1% in 1991 to 5.8% in 2004. However, it tended to rise again in the 2006 round and reached 7.3% in 2015. The trends of overweight and obesity continued to rise from 1991 to 2015 on a yearly basis, from 24.6% to 38.2%, and from 2.5% to 12.6%, respectively (Figure 1). Of note, the proportion of women who had normal BMI has declined monotonically from 62.8% in 1991 to 41.9% in 2015.

Table 1 summarizes the distribution of various characteristics of women of reproductive age across four categories of malnutrition from the CHNS 1991–2015. The average age of the participants was 35.2 years, and 66.7% were rural residents. Furthermore, 72.8% of the total population didn’t have a high school diploma, and 52.8% were not insured. Finally, 98.2% and 89.9% of women of reproductive age never smoked or had a drinking habit, respectively.

### 3.2. Individual and Household Effects

Age was significantly associated with nutritional status of women, showing that older women were more likely to suffer from overweight or obesity, and younger women were more likely to be underweight. Marital status was also different across categories, with single status noted among 38.5% underweight reproductive women, which dropped to 3.9% in women who were obese. Education and household economic status were also important predictors of women’s nutritional status.

Table 2 presents the estimated adjusted odds ratios (ORs) with their 95% CIs from multinomial logit models on malnutrition with four scenarios (using normal BMI as the reference) for women of reproductive age in China. Married women appeared to be at increased odds of overweight and obesity by 23.8% (95% CI: 9.7 to 39.8) and 46% (10.8 to 92.3) respectively, but were also at decreased odds of underweight by 29.9% compared to unmarried women (95% CI: 0.602 to 0.817). Divorce/widowhood/separation status also increased the odds of obesity by 65.6% (95% CI: 9.6 to 150.4).

Education level was a protective factor of women overweight and obesity. The odds of overnutrition decreased when women received more formal schooling. Specifically, the odds of suffering from overweight were reduced by an estimated 24.5% (95% CI: 66.8 to 85.4) among high school or vocational school graduates, and 33.5% (95% CI: 47.5 to 67.2) among women with a university degree, compared to illiterates or primary school dropouts; moreover, the odds of obesity were reduced by 52.4% (95% CI: 38.0 to 59.5) and 72.2% (95% CI: 20.1 to 38.5), respectively. The lowest and highest income household income per capita did not appear to increase the odds of overweight/obesity among women of reproductive age. The odds of overweight, however, increased by 11.1% (95% CI: 2.0 to 20.9) for women from medium-income families.

### 3.3. Community Socioeconomic Factors and Time Effects

Urban residency was not a preventive factor of underweight. It was associated with higher odds of overweight and obesity by 22.3% (95% CI: 7.2 to 39.5) and 33.9% (95% CI: 2.7 to 74.6), respectively, which acted as another significant predictor of overweight/obesity among women of reproductive age. Women from the medium urbanized communities tended to have higher odds of overweight and obesity than the other two groups. The medium level of urbanization was associated with a substantial increase of 18.5% (95% CI: 7.5 to 30.5) in the odds of overweight, and tended to be associated with increased odds of obesity by 40.4% (95% CI: 17.0 to 68.6). Women from less urbanized areas tended to have higher odds of undernutrition. However, urbanization did not appear to affect the likelihood of underweight, and the highest level was not associated with an increase or reduction of odds of abnormal BMI.

There were substantial regional variations in malnutrition of women of reproductive age. The southwest area, the most deprived of the survey regions, had the highest prevalence of underweight and the lowest overnutrition prevalence. The northeast region and the coastal area had a higher prevalence of overweight/obesity compared to the other two regions. Compared to the most deprived southwest area, women from the northeast, central, and coastal areas were less likely to be underweight (OR = 0.642, 95% CI: 0.503 to 0.819; OR = 0.620, 95% CI: 0.504 to 0.761; OR = 0. 528, 95% CI: 0.418 to 0.665, respectively). The odds of reproductive-aged overweight or obesity increased 33.4% (95% CI: 13.7 to 56.6) and 78.2% (95% CI: 27.7 to 148.6), respectively, in the central area; 63.8% (95% CI: 36.2 to 97.1) and 187.9% (95% CI: 97.0 to 32.5),respectively, in the northeast area; and 59.8% (95% CI: 34.4 to 190.0) and 137.9% (95% CI: 66.9 to 239.1), respectively, in the coastal area. The odds of overweight/obesity among women of reproductive age appeared to increase over time. Furthermore, the risk of being underweight tended to increase after 2006 compared to the reference 1991.

## 4. Discussion

This study examined trends, patterns, distributions, and underlying mechanisms of four exclusive nutrition statuses among women of reproductive age in China. The results indicated that household/individual level sociodemographic and socioeconomic factors, including marital status, education level, medical insurance, and household income per capita, are significant predictors of women’s nutritional status. This study also highlighted the importance of urbanization index, residence region, and the temporal effects, which play a role in malnutrition of women of reproductive age.

Individual socioeconomic factors are still major determinants that influence women’s nutritional status. Our results indicate that women with higher education level may have stronger health awareness and diet management than those with lower education level, which can prevent overnutrition. Literacy and numeracy skills gained from school education enable women to obtain health-related knowledge, regarding micronutrients and balanced diets, as well as stronger health awareness. We found that older women and women who were currently or previously married were more likely to be overweight/obesity than younger or single women. This may be explained by the fact that married couples might not be worried whether they will be attractive to a partner if they are overweight [25]. Little time available for household chores or active leisure as well as postpartum weight gain were also causes for their overnutrition status [25,26,27]. Moreover, as also mentioned by other researchers, the nutritional status of married women may be associated with the partner’s education level [28]. Additionally, the status of a divorced/separated woman or a widow leads to the occurrence of stress under Chinese traditional culture, which may have contributed to the status of high BMI [29]. The increased risk of overweight/obesity as age increased could also be explained by limited opportunities for exercise due to the numerous family duties and stress associated with daily work [25].

The increased purchasing power among many families in China together with a booming economic market result in the influx of high-calorie product exposure for income-improved families. However, from this study we observed that medium rather than the highest household income was associated with increased risk of overweight among women of reproductive age. We postulate that women in the highest-income families have greater access to knowledge and are better equipped with information processing skills. The media advertisements might persuade women from medium-income families to a relatively greater degree. The potential interactions between education and wealth on obesity may lead to the uncertainty of this result [30], and further studies focusing on these two factors are warranted.

Our results show that improved community urbanization level, including living in more urbanized communities, and being a resident in urban or more developed provinces reduce the risk of overnutrition among Chinese women of reproductive age. Women from medium-urbanized communities, instead of from the most urbanized ones, are at increased risk of being overweight. It is not consistent with the existing evidence that the prevalence of overweight/obesity increases with the degree of urbanization for both the genders in China [31]. Aside from improved economic status and purchasing power for high calorie-dense foods, obesity and overweight are also often driven by certain contextual factors such as dietary habits and obesogenic physical inactivity environments [32,33]. Rapid environmental, economic, and social changes that follow urbanization may put people in risky situations such as environmental hazards, stressors, and unhealthy diets and lifestyles [34,35]. The dietary transition has entered a new stage in China since 1990, with increasing dietary energy density and a sharp decrease in coarse grains and dietary fiber intake. Intake of vegetables and fruits was also inadequate. The same trend is also notable in Mexico, Egypt, Thailand, and other developing countries [36,37]. This may be partly due to poor nutritional knowledge and dietary preferences, as well as the food choices available within neighborhoods. Under these circumstances, residents in the most urbanized regions are exposed to sources of information about healthy foods and health-related education. Communities of medium urbanization may not have the same benefits that enable diet adequacy and quality [38,39].

Given the co-existence of underweight and overweight in many communities, double-duty integrated interventions are critically needed. In line with the trend in most low-income and middle-income countries [40], we, and other researchers, note that China is experiencing a double burden of malnutrition among adults by adding obesity and related chronic diseases to the public health agenda, while previous nutritional problems of undernutrition still remain [41,42]. Along with the rising prevalence of overweight and obesity, the prevalence of underweight among women of reproductive age has been reduced to some extent since 1991 [43]. It has plateaued since 1997, with a suggestion of increased risk in more recent years. Socioeconomic factors may provide a possible explanation for this finding as inadequate food, or nutrient deficiency, is often prevalent in deprived economic conditions. Moreover, substantial regional disparities found in our study are critical to note with the highest risk in southwest China and the lowest in the coastal area.

Different forms of malnutrition may be influenced by shared drivers, including biological, socioeconomic, and environmental, and also could be addressed through shared platforms. The potential platforms indicated by the WHO are national dietary guidelines, health systems, humanitarian aid and emergency nutrition programs, national-level policies for obesity, non-communicable diseases (NCDs) and nutrition, urban food policies, and social policies [44]. Folic acid and iron supplementation are emphasized, as well as preconception nutritional counselling, to provide adequate and accurate knowledge the foods, and in what quantities, are required for optimal intake [44]. The economic effects of these double-duty interventions have also been proven by researchers [45]. The policy implications from our study include the need for increased attention to improving the community environment, levels of education, and living environment. In order to improve the coverage of primary food markets that usually sell healthy fresh food, as well as the accessibility of health education, increasing the investment in food availability and strengthening the construction of health-related infrastructure in economically backward areas should be put on the agenda. However, the training of health workers also matters. They are key figures to intervene in maternal health in Chinese rural regions and areas. In addition, overweight/obesity-targeted lectures (or other education patterns) or activities must be held in middle-urbanized communities, as well as neighborhoods largely covered by middle-income families. Short-term intervention is not enough to narrow the gap between developed and developing areas. Long-term and sustainable plans for nutrition improvement should be made in order to avoid as much as possible the rebound of nutrition promotion affected by other factors.

Some methodological limitations warrant cautious interpretation of our findings. First, this study uses BMI status to represent the status of malnutrition among women of reproductive age. Since relevant data were not included in the CHNS, this study did not take other indicators, such as weight loss or micronutrient deficiencies, into account. Second, nutrition demand for women of reproductive age can vary significantly in different periods. Women who are in preparation for pregnancy or postpartum recovery may adopt a specific dietary structure or physical activity mode, which might result in a particular BMI pattern. We were unable to examine this specificity given the unavailability of this kind of data from the CHNS. Future studies can separate women of reproductive age into different period groups in order to reduce this effect among the women populations.

## 5. Conclusions

Due to the importance of yet neglected awareness of the nutritional status and relevant determinants among women of reproductive age, more targeted interventions should be implemented according to evidence-based analysis. In conclusion, our findings highlight that a mixture of risk factors from the individual, household, and community levels play critical roles in under- and overnutrition among women of reproductive age in China. Improving nutrition-related education and household environment as well as the living environment from a wider context in economically backward areas are essential to enhance women’s undernutrition status. Greater attention is needed to stem the rising tide of overweight and obesity among women of reproductive age from middle-income families or medium-urbanized communities.

## Figures and Tables

**Table 1:** Distribution of women of reproductive age across four categories of malnutrition by different predictors from the China Health and Nutrition Survey (CHNS) 1991–2015.

Variables	Total	Underweight	Normal	Overweight	Obese	*p*-Value
Sample size (*n* (%))	24,606	1926 (7.8)	13,209 (53.7)	7697(31.3)	1774 (7.2)	
Age (years), mean (SD)	35.2 (8.8)	30.0 (9.9)	33.6 (9.3)	38.3 (7.9)	40.0 (7.0)	<0.0001
Marriage status (*n* (%))						<0.0001
Single	3950 (16.1)	742 (38.5)	2579 (19.5)	560 (7.3)	69 (3.9)	
Married	20,093 (81.7)	1146 (59.5)	10,335 (78.2)	6965 (90.5)	1647 (92.8)	
Divorced/widowed/separated	563 (2.3)	38 (1.96)	295 (2.2)	172 (2.2)	58 (3.3)	
Education (*n* (%))						<0.0001
Illiterate or primary	3816 (15.5)	205 (10.6)	2004 (15.2)	1314 (17.1)	293 (16.5)	
Primary graduates or junior school	14,109 (57.3)	1083 (56.2)	7412 (56.1)	4501 (58.5)	1113 (62.7)	
High school or vocational school	4919 (20.0)	449 (23.3)	2770 (21.0)	1420 (18.5)	280 (15.8)	
University or above	1762 (7.2)	189 (9.8)	1023 (7.7)	462 (6.0)	88 (4.94)	
Nationality (*n* (%))						<0.0001
Han	21,599 (87.8)	1653 (85.8)	11,512 (87.2)	6850 (89.0)	1584 (89.3)	
Others	3007 (12.2)	273 (14.2)	1697 (12.8)	847 (11.0)	190 (10.7)	
Medical insurance (*n* (%))						<0.0001
No	12,985 (52.8)	1127 (58.5)	7543 (57.1)	3630 (47.2)	685 (38.6)	
Yes	11,621 (47.2)	799 (41.5)	5666 (42.9)	4067 (52.8)	1089 (61.4)	
Household income per capita (*n* (%))						<0.0001
Lowest	8310 (33.8)	753 (39.1)	4951 (37.5)	2207 (28.7)	399 (22.5)	
Middle	9022 (36.7)	652 (33.9)	4726 (35.8)	2965 (38.5)	679 (38.3)	
Highest	7274 (29.6)	521 (27.1)	3532 (26.7)	2525 (32.8)	696 (39.2)	
Residency (*n* (%))						<.0001
Urban	8185 (33.3)	690 (35.8)	4239 (32.1)	2663 (34.6)	593 (33.4)	
Rural	16,421 (66.7)	1236 (64.2)	8970 (67.9)	5034 (65.4)	1181 (66.6)	
Region (*n* (%))						<0.0001
Southwest	6087 (24.7)	716 (37.2)	3546 (26.9)	1543 (20.1)	282 (15.9)	
Northeast	4368 (17.8)	264 (13.7)	2109 (16.0)	1541 (20.0)	454 (25.6)	
Central	8451 (34.4)	617 (32.0)	4680 (35.4)	2618 (34.0)	536 (30.2)	
Coastal	5700 (23.2)	329 (17.1)	2874 (21.8)	1995 (25.9)	502 (28.3)	
Urban index (*n* (%))						<0.0001
Lowest	8757 (35.6)	701 (36.4)	5124 (38.8)	2468 (32.1)	464 (26.2)	
Middle	8119 (33.0)	624 (32.4)	4128 (31.3)	2683 (34.9)	684 (38.6)	
Highest	7730 (31.4)	601 (31.2)	3957 (30.0)	2546 (33.1)	626 (35.3)	
Ever smoked (*n* (%))						0.0013
No	24,154 (98.2)	1892 (98.2)	13,000 (98.4)	7537 (97.9)	1725 (97.2)	
Yes	452 (1.8)	34 (1.8)	209 (1.6)	160 (2.1)	49 (2.8)	
Drinking (*n* (%))						0.1498
Never	22,122 (89.9)	1755 (91.1)	11,891 (90.0)	6883 (89.4)	1593 (89.8)	
Yes	2484 (10.1)	171 (8.9)	1318 (10.0)	814 (10.6)	181 (10.2)	
Wave (*n* (%))						<0.0001
1991	3298 (13.4)	334 (17.3)	2071 (15.7)	810 (10.5)	83 (4.7)	
1993	3068 (12.5)	317 (16.5)	1869 (14.2)	782 (10.2)	100 (5.6)	
1997	2893 (11.8)	219 (11.4)	1714 (13.0)	809 (10.5)	151 (8.5)	
2000	2939 (11.9)	235 (12.2)	1548 (11.7)	952 (12.4)	204 (11.5)	
2004	2505 (10.2)	144 (7.5)	1304 (9.9)	856 (11.1)	201 (11.3)	
2006	2354 (9.6)	146 (7.6)	1217 (9.2)	796 (10.3)	195 (11.0)	
2009	2324 (9.4)	174 (9.0)	1156 (8.8)	773 (10.0)	221 (12.5)	
2011	3036 (12.3)	197 (10.2)	1413 (10.7)	1082 (14.1)	344 (19.4)	
2015	2189 (8.9)	160 (8.3)	917 (6.9)	837 (10.9)	275 (15.5)	

Chi-squared test or ANOVA for a cross-tabulation between each variable and the four categories of malnutrition.

**Table 2:** Adjusted odds ratio (OR) and 95% confidence interval (CI) for predictors of unordered multinomial logistic models (using normal BMI as the reference) predicting malnutrition for women of reproductive age from the CHNS, 1991–2015.

Variable	BMI Level (Reference: Normal BMI)					
	Underweight		Overweight		Obesity	
	Adjusted OR (95% CI)	*p*-Value	Adjusted OR (95% CI)	*p*-Value	Adjusted OR (95% CI)	*p*-Value
**Individual Level**						
Age (years)	0.955 (0.947–0.962)	<0.0001	1.057 (1.052–1.061)	<0.0001	1.074 (1.065–1.083)	<0.0001
Marital status (ref: single)						
Married	0.701 (0.602–0.817)	<0.0001	1.238 (1.097–1.398)	0.0007	1.460 (1.108–1.923)	0.009
Divorced/widowed/separated	0.828 (0.568–1.208)	0.34	0.991 (0.785–1.250)	0.92	1.656 (1.096–2.504)	0.02
Education (ref: illiterate or primary)						
Primary graduates or junior	0.947 (0.791–1.133)	0.53	1.024 (0.930–1.127)	0.58	0.863 (0.728–1.023)	0.09
High school or vocational	1.056 (0.852–1.309)	0.57	0.755 (0.668–0.854)	<0.0001	0.476 (0.380–0.595)	<0.0001
University or above	1.12 (0.843–1.446)	0.41	0.565 (0.475–0.672)	<0.0001	0.278 (0.201–0.385)	<0.0001
Nationality (ref: Han)						
Others	0.906 (0.742–1.107)	0.33	1.050 (0.918–1.200)	0.46	1.039 (0.818–1.321)	0.75
Medical insurance (ref: no)						
Yes	0.960 (0.829–1.112)	0.54	1.127 (1.033–1.229)	0.002	1.077 (0.920–1.261)	0.38
**Household Income per Capita (Ref: Lowest)**						
Middle	0.988 (0.859–1.137)	0.80	1.111 (1.020–1.209)	0.01	1.004 (0.861–1.172)	0.98
Highest	1.095 (0.902–1.328)	0.40	1.009 (0.903–1.128)	0.80	0.837 (0.694–1.009)	0.06
Ever smoked (ref: yes)						
Yes	1.686 (1.144–2.484)	0.009	0.952 (0.760–1.193)	0.69	1.094 (0.772–1.551)	0.58
Drinking (ref: never)						
Yes	0.901 (0.754–1.076)	0.27	1.011 (0.913–1.120)	0.82	1.070 (0.890–1.287)	0.53
**Community Level and Time Effect**						
Residency (ref: rural)						
Urban	1.059 (0.885–1.267)	0.52	1.223 (1.072–1.395)	0.002	1.339 (1.027–1.746)	0.01
Region (ref: southwest)						
Central	0.620 (0.504–0.761)	<0.0001	1.334 (1.137–1.566)	0.0001	1.782 (1.277–2.486)	0.001
Coastal	0.528 (0.418–0.665)	<0.0001	1.598 (1.344–1.900)	<0.0001	2.379 (1.669–3.391)	<0.0001
Northeast	0.642 (0.503–0.819)	0.0004	1.638 (1.362–1.971)	<0.0001	2.879 (1.970–4.205)	<0.0001
Urban index (ref: lowest)						
Middle	1.072 (0.920–1.249)	0.42	1.185 (1.075–1.305)	0.001	1.404 (1.17–1.686)	<0.0001
Highest	1.144 (0.923–1.418)	0.25	1.029 (0.897–1.182)	0.65	1.137 (0.877–1.473)	0.35
Wave (ref: 1991)						
1993	1.058 (0.891–1.257)	0.40	1.011 (0.894–1.143)	0.84	1.196 (0.888–1.613)	0.34
1997	0.825 (0.673–1.010)	0.06	1.061 (0.929–1.211)	0.39	1.973 (1.472–2.644)	<0.0001
2000	0.999 (0.811–1.231)	1.00	1.364 (1.192–1.561)	<0.0001	2.891 (2.169–3.854)	<0.0001
2004	0.842 (0.660–1.074)	0.18	1.293 (1.120–1.493)	0.000	2.995 (2.220–4.041)	<0.0001
2006	0.929 (0.727–1.189)	0.61	1.236 (1.066–1.433)	0.005	3.105 (2.288–4.213)	<0.0001
2009	1.199 (0.926–1.553)	0.16	1.169 (0.994–1.375)	0.06	3.625 (2.627–5.001)	<0.0001
2011	1.101 (0.843–1.437)	0.46	1.443 (1.227–1.696)	<0.0001	5.256 (3.820–7.230)	<0.0001
2015	1.319 (0.991–1.756)	0.07	1.815 (1.523–2.164)	<0.0001	7.349 (5.247–10.295)	<0.0001
